# Facial Structure Alterations and Abnormalities of the Paranasal Sinuses on Multidetector Computed Tomography Scans of Patients with Treated Mucosal Leishmaniasis

**DOI:** 10.1371/journal.pntd.0003001

**Published:** 2014-07-31

**Authors:** Raphael Abegão de Camargo, Antonio C. Nicodemo, Daniel Vaccaro Sumi, Eloisa Maria Mello Santiago Gebrim, Felipe Francisco Tuon, Lázaro Manoel de Camargo, Rui Imamura, Valdir Sabbaga Amato

**Affiliations:** 1 Department of Infectious Diseases, University of São Paulo Medical School, São Paulo, São Paulo, Brazil; 2 Institute of Radiology, Hospital das Clínicas, University of São Paulo Medical School, São Paulo, São Paulo, Brazil; 3 Division of Infectious Diseases, Federal University of Paraná, Curitiba, Paraná, Brazil; 4 Division of Infectious and Parasitic Diseases, Hospital Universitário Evangélico de Curitiba, Curitiba, Paraná, Brazil; 5 University of Cuiabá, Veterinary Medical School, Cuiabá, Mato Grosso, Brazil; 6 Department of Otorhinolaryngology and Ophthalmology, University of São Paulo Medical School, São Paulo, São Paulo, Brazil; New York University, United States of America

## Abstract

**Background/Objectives:**

Mucosal leishmaniasis (ML) is a progressive disease that affects cartilage and bone structures of the nose and other upper respiratory tract structures. Complications associated with ML have been described, but there is a lack of studies that evaluate the structural changes of the nose and paranasal sinuses in ML using radiological methods. In this study, we aimed to assess the opacification of the paranasal sinuses in patients with treated ML and any anatomical changes in the face associated with ML using multidetector computed tomography scans (MDCT) of the sinuses. We compared the findings with a control group.

**Methodology/Principal Findings:**

We evaluated 54 patients with treated ML who underwent CT scans of the sinuses and compared them with a control group of 40 patients who underwent orbital CT scans. The degree of sinus disease was assessed according to the Lund-Mackay criteria. Forty of the 54 patients with a history of ML (74.1%) had a tomographic score compatible with chronic sinusitis (Lund-Mackay ≥4). CT scans in the leishmaniasis and control groups demonstrated significant differences in terms of facial structure alterations. Patients from the ML group showed more severe levels of partial opacification and pansinus mucosal thickening (42.6%) and a greater severity of total opacification. Patients from the ML group with a Lund-Mackay score ≥4 presented longer durations of disease before treatment and more severe presentations of the disease at diagnosis.

**Conclusion/Significance:**

CT scans of the sinuses of patients with ML presented several structural alterations, revealing a prominent destructive feature of the disease. The higher prevalence in this study of chronic rhinosinusitis observed in CT scans of patients with treated ML than in those of the control group suggests that ML can be considered a risk factor for chronic rhinosinusitis in this population (p<0.05).

## Author Summary

Mucosal leishmaniasis (ML) is mainly caused by the agent *Leishmania (V.) brasiliensis* and usually occurs months or years after symptomatic or asymptomatic skin infection. Approximately 5% of patients with untreated cutaneous leishmaniasis will develop ML, a presentation that causes significant morbidity in patients. This important but often neglected anthropozoonosis also presents direct health care costs and several indirect losses in terms of impaired daily functioning. In this study, we aimed to assess the degree of opacification of the paranasal sinuses in patients with treated ML and any anatomical changes in the face associated with ML using multidetector computed tomography scans (MDCT) of the sinuses. We compared the findings in this population with those from a control group. We evaluated 54 patients with treated ML who were subjected to MDCT of the sinuses and compared them with a control group of 40 patients who underwent MDCT of the orbit. CT scans of the sinus of patients with ML presented several structural alterations, revealing a prominent destructive feature of the disease. The higher prevalence of chronic rhinosinusitis observed in CT scans of patients with treated ML in this study also suggests that ML can be considered a risk factor for chronic rhinosinusitis in this population.

## Introduction

Leishmaniasis comprises a complex spectrum of diseases with clinical relevance and epidemiological diversity. *Leishmania* infections are globally distributed; they occur on five continents and represent a serious public health problem in each. It is estimated that there are currently 12 million cases. The World Health Organization estimates that 350 million people in 88 countries around the world are at risk, and approximately two million new cases with varying clinical manifestations are recorded annually [1]–[3]. Of these, 1.5 million and 500,000 cases correspond to tegumentary and visceral leishmaniasis, respectively [3].

*Leishmania* is an obligate intracellular parasite transmitted by phlebotomus sandflies, and depending on the characteristics of the host and the infecting *Leishmania* species, local or systemic inflammation may develop. Leishmaniasis can be classified as visceral or tegumentary. Visceral leishmaniasis causes a chronic disease that is characterized by impairment of the reticuloendothelial system, which leads to hepatomegaly and splenomegaly associated with pancytopenia and generates a systemic condition that can lead to death. Tegumentary leishmaniasis tends to be a localized disease, although the diffuse involvement of skin and mucous membranes may also occur. Due to these varying clinical aspects of the disease, tegumentary leishmaniasis can be divided into localized cutaneous leishmaniasis, diffuse cutaneous leishmaniasis and mucosal leishmaniasis [1], [3].

Tegumentary leishmaniasis is still classified by region as Old World leishmaniasis (i.e., southern Europe, the Middle East, Asia and Africa) and New World leishmaniasis (i.e., Latin America), which is also described as American tegumentary leishmaniasis (ATL) [1], [3], [4]. While most of the Old World species cause benign cutaneous disease, the species associated with New World leishmaniasis usually cause a spectrum of disease ranging from minor skin lesions to severe mucosal lesions [4].

Mucosal leishmaniasis (ML) is mainly caused by *Leishmania braziliensis*, and it occurs months or years after the cutaneous lesions have healed. Approximately 5% of patients with the cutaneous form of the disease will develop ML, the most severe presentation of ATL, which can cause progressive destruction of soft tissue cartilage and bone structures of the nose and other upper respiratory tract structures often culminating with septal perforation [5], [6]. Nasal involvement can be responsible for changes in the drainage of the paranasal sinuses, leading to chronic sinusitis in addition to late facial disfigurement due to the destruction of the nasal septum [7]. In more severe cases that involve the pharynx and/or larynx, aspiration or airway obstruction can be a life-threatening consequence for the patient [8], [9].

Though the most common complaints reported by patients with ML include nasal obstruction, rhinorrhea, epistaxis and posterior drainage [10], [11], little is known about the prevalence of chronic sinus disease in the population with ML. There is also a lack of descriptive studies on the structural anatomical changes of the nose and paranasal sinuses using radiological methods in patients with ML [7]. Therefore, we aimed to determine the prevalence of chronic sinusitis using computed tomography (CT) of the sinuses in patients with ML post-treatment and to identify the existence of any tomographic changes in the nose and paranasal sinuses that may be related to ML.

## Methods

### Patients

**Mucosal leishmaniasis group.** This prospective study evaluated the facial anatomy of 54 patients who had a confirmed diagnosis of ML and met the criteria for cure after treatment using Multidetector Computed Tomography scans (MDCT) of the paranasal sinuses. The diagnosis of ML was determined by conclusive biopsy of the nasal mucosa (i.e., presence of the amastigote form of *Leishmania* visualized by staining with hematoxylin/eosin, presence of *Leishmania* antigens based on immunohistochemical analysis or demonstration of parasite DNA by Polymerase Chain Reaction), and the criteria for cure after treatment was defined by complete regression of mucosal lesions, determined by clinical examination and nasal endoscopy one year after the end of treatment at the Leishmaniasis Ambulatory of the Hospital das Clínicas (University of São Paulo School of Medicine) from December 2008 to November 2011. No patient had a history of rhinosinusitis, allergic rhinitis or sinus surgery, and no patients presented symptoms of infection of the upper airways on the day of the CT scan [12]. **Control group.** A series of patients without a clinical diagnosis of ML who underwent MDCT of the orbit in 2009 and 2010 was extracted from a radiological examination database. The indications for performing the orbit CT included orbit infection, optic neuritis, proptosis, lacrimal disorders, Graves ophthalmopathy, orbital tumors and pseudotumors, assessment of eye trauma and evaluation of progressive vision loss and decreased range of eye motion. Patients who had a history of trauma with fracture or surgery of the skull, nose or paranasal sinuses and those whose MDCT of the orbit did not show all of the paranasal sinuses were excluded from the study [13]. In addition, the electronic medical record for each patient was reviewed, and patients who were evaluated in the otolaryngology division for any diagnoses of sinus disease were excluded from subsequent analysis. We opted to select patients who underwent computed tomography of the orbit because patients submitted to specific tomography of the paranasal sinuses would most likely have or be suspected to have a disease of the rhinosinusal system that justified the test request [13], [14]. Additionally, unnecessary computed tomography radiation exposure of patients without nasosinusal disease for the sole purpose of forming a control group would be ethically inappropriate. A total of 40 patients with sex and age distributions similar to the mucosal leishmaniasis group were selected from the 269 patients who met the eligibility criteria.

### Ethics statement

This study was approved by the Ethics Board of the Hospital das Clínicas of the University of São Paulo School of Medicine, São Paulo, Brazil. All patients in this study were followed up according to the standard protocol established by the leishmaniasis service unit, with no active search. The project was approved by the Ethics Committee for Analysis of Research Projects (CAPPesq) of the University of São Paulo Medical School under protocol number 0469/07.

The present study used CT scans of the paranasal sinuses of patients enrolled in a protocol developed by the Division of Infectious and Parasitic Diseases of the University of São Paulo Medical School, and all patients were requested to sign an informed consent form prior to the CT scans. On this form, appropriate explanations and guidelines about the procedure were addressed using accessible language aimed at generating understanding in the patients and their companions.

### Clinical findings

We obtained demographic, epidemiologic and clinical data about the ML group regarding age, gender, race, comorbidities (i.e., high blood pressure, diabetes, chronic renal failure and HIV infection), symptoms at admission (i.e., nasal obstruction, epistaxis, rhinorrhea, odynophagia, itching, facial pain, headache, hyposmia, dysphagia, hoarseness and dyspnea), lesion site (i.e., septum, palate and larynx), duration of disease before treatment (<1 year, 1–2 years and >2 years) and severity of disease. Disease severity was assessed by combining the criteria regarding mucosal lesion extent with the severity of the symptoms (i.e., (1) mild, those symptoms confined to the nose; (2) moderate, involvement of two or more mucous membranes with mild or no respiratory distress; and (3) severe, moderate disease plus severe respiratory distress) [15].

### Radiologic aspects

All patients underwent CT scans of the paranasal sinuses (ML group) or orbits (control group). The CT scans were performed using a 16- or 64-channel multidetector scanner (IDT16 and Brilliance 64, Philips Medical Systems, Amsterdam, Netherlands) without contrast media. The following parameters were used: 120 kV, 100 mA and a rotation time of 0.75 s in the axial plane, a slice thickness of 1 mm and an increment of 0.5 mm on bone and standard algorithms of reconstruction. Extra-sinusal abnormalities were observed in nine patients, and in these cases, another sequence was performed using intravenous contrast media (iobitridol, Henetix 300, Guerbet) at a dose of 1.5 ml/kg.

**Figure 1:**
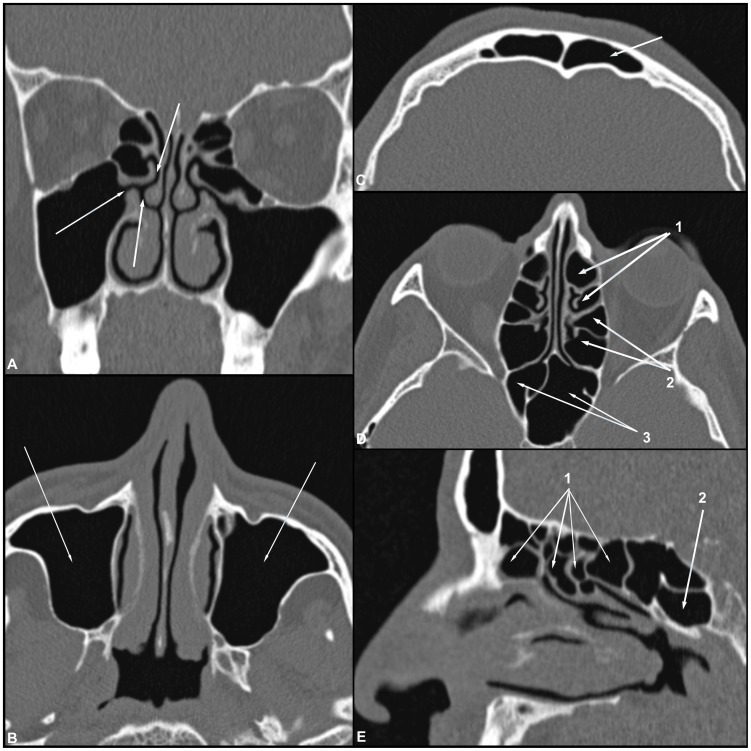
(A–E) Coronal, axial and sagittal CT images of the bone window demonstrate the normal anatomy of the paranasal sinuses and the integrity of the nasal septum, nasal bone and nasal pyramid. Ostiomeatal complex (A). Maxillary sinus (B). Frontal sinus (C). Anterior ethmoid air cells (arrow 1), posterior ethmoid air cells (arrow 2) and sphenoid sinuses (arrow 3) (D). Ethmoid air cells (arrow 1) and sphenoid sinuses (arrow 2) in sagittal slice (E).

The volumetric data were transferred to a workstation, and multiplanar reconstructions (i.e., axial, coronal and sagittal planes) were obtained. All CT scans were analyzed by a radiologist who was unaware of the clinical condition of the patient (i.e., ML or control). The radiologist evaluated the degree of opacification of the paranasal sinuses (sinusopathy) and ostiomeatal complexes (Figure 1) as well as the presence of any abnormality that could be related to leishmaniasis, such as foci of erosion in the nasal septum, deformity of the nasal pyramid and cutaneous and subcutaneous and mucosal lesions.

Sinusopathy was graded according to the Lund-MacKay criteria, in which scores are established according to the degree of opacification for each sinusal system and for the ostiomeatal complexes based on their appearance on the CT scan [16]–[19]. If inflammation covers 0% of the image, a score of 0 is assigned, and when it covers 100% of the image, a score of 2 is assigned. Any degree of partial coverage is scored as 1. To subdivide the extension of inflammation in grade 1, partial opacification was further stratified into three categories (i.e., 1A, from 1% to 33%; 1B from 34% to 66%; and 1C, from 67% to 99%) according to the modification to the Lund-Mackay system proposed by Meltzer et al. (2004) [20], [21]. Due to the previously established high inter-rater reliability of the Lund-Mackay paranasal sinus staging system, multiple reviewers were not used in this study [22].

### Comparative analysis

All tomographic findings in the ML and control groups were compared, along with the estimated grade of sinusopathy, according to the Lund-Mackay system. Data related to the ML group were further analyzed by dividing the 54 patients into 2 groups according to the presence (Lund-Mackay ≥4) or absence (Lund-Mackay score <4) of sinus disease; the groups had 40 and 14 patients, respectively. Considering that incidental abnormalities are commonly observed in asymptomatic individuals [13], [14], [23], a Lund-Mackay score greater than or equal to four has been defined as the gold standard cut-off for chronic rhinosinusitis [13], [24], [25]. Exploratory univariate analyses were performed to identify predictive variables associated with a greater severity of sinusopathy in this population.

### Statistical analysis

Categorical variables were descriptively presented in tables containing absolute (n) and relative (%) values. The association between them was assessed using a chi-square test, a likelihood ratio or Fisher's exact test. The normality of the quantitative variables was evaluated using the Kolmogorov-Smirnov test. Quantitative variables with normal distributions were presented descriptively in tables containing the means and standard deviations. The averages of these variables were compared using Student's t-test. Quantitative variables that did not follow a normal distribution were presented descriptively in tables containing the median and interquartile ranges. The distributions of these variables were compared using a Mann-Whitney test. P values<0.05 were considered statistically significant. A multiple regression model explored relevant predictors of the severity of sinusopathy in the ML group, with the Lund-Mackay score as the dependent variable. The variables gender, erosion of the septum and nasal bone, collapse of the nasal pyramid, alteration of the nasal conchae, number of previous treatments, time of disease, severity of the leishmaniasis disease, nasal obstruction, rhinorrhea, epistaxis, hyposmia and dyspnea were tested as predictors using a stepwise method. The calculations were processed using SPSS software version 18.0 (SPSS Inc, Chicago, Illinois).

## Results

### General data

The mean age of patients with ML in this study was 60±13 (range 25–85) years. Sixty-three percent of patients were male, and 50% were Caucasian. In the control group, the mean age was 55±15 (range 28–90) years, and 62.5% of patients were male. Most patients in the control group were Caucasian (67.5%) (Table 1).

**Table 1:** Demographic data from 54 patients with Mucosal Leishmaniasis and 40 patients in the control group.

Characteristic	Leishmaniasis (n = 54)		Control (n = 40)		P
**Age (years)**	60±13		55±15		0.094^1^
**Sex (Male)**	34	63.0%	25	62.5%	0.963^2^
**Origin (region)**					
Northeast	25	46.3%	8	20.0%	0.001^3^
Southeast	17	31.5%	29	72.5%	
Center-west	3	5.6%	0	0.0%	
South	7	13.0%	2	5.0%	
North	2	3.7%	1	2.5%	
**Race**					
Caucasian	27	50.0%	27	67.5%	<0.001^3^
Mixed race	19	35.2%	4	10.0%	
African descent	8	14.8%	2	5.0%	
Asian	0	0.0%	7	17.5%	

1 - Student's t-test.

2 - Pearson Chi-square.

3 - Likelihood ratio.

### Clinical findings

The most common symptoms in the ML group were nasal obstruction (53/54, 98.1%), epistaxis (41/54, 75.9%) and rhinorrhea (28/54, 51.9%); high blood pressure was the most common comorbidity (32/54, 59.3%) (Table 2).

**Table 2:** Symptoms and comorbidities of patients with mucosal leishmaniasis.

Characteristic	Leishmaniasis	Prevalence	
	(n = 54)	(%)	95%CI
**Symptoms**			
Nasal obstruction	53	98.1	90.1–99.9
Epistaxis	41	75.9	62.4–86.5
Rhinorrhea	28	51.9	37.8–65.7
Odynophagia	20	37.0	24.3–51.3
Coryza	12	22.2	12.0–35.6
Hyposmia	8	14.8	6.6–27.1
Itching	7	13.0	5.4–24.9
Hoarseness	6	11.1	4.2–22.6
Dyspnea	3	5.6	1.2–15.4
Facial pain	3	5.6	1.2–15.4
Headache	2	3.7	0.5–12.8
**Comorbities**			
Hypertension	32	59.3	45.0–72.4
Chronic renal failure	4	7.4	2.1–17.9
Diabetes	3	5.6	1.2–15.4
HIV infection	2	3.7	0.5–12.8

### General radiological findings

CT scans in the ML and control groups demonstrated significant differences in terms of facial structure alterations (Table 3). Patients from the ML group showed more severe levels of partial opacification (1B + 1C) and pansinus mucosal thickening (23/54, 42.6%) and a greater severity of total opacification (Table 4). In general, patients from this group presented a higher median Lund-Mackay score (7, p<0.001) than did patients from the control group (3, p<0.001).

**Table 3:** Radiological findings from the leishmaniasis and control groups.

Characteristics	Leishmaniasis			Control			P
	(n = 54)			(n = 40)			
Structural findings from CT scan	n	%	95%CI	n	%	95%CI	
Erosion of the nasal septum	36	66.7%	52.53–78.91%	0	0.0%	0–8.8%*	<0.001^1^
Collapse of the nasal pyramid	8	14.8%	6.62–27.12%	0	0.0%	0–8.8%*	0.019^2^
Alterations of the nasal conchae	21	38.9%	25.92–53.12%	2	5.0%	0.61–16.92%	<0.001^1^
Retention cysts/polyp of the maxillary sinus	15	27.8%	16.46–41.64%	14	35.0%	7.54–20.63%	0.4541
Osteitis of the paranasal sinus	13	24.1%	13.49–37.64%	2	5.0%	0.61–16.92%	0.013^1^
Thickening of the nasal cavity	21	38.9%	25.92–53.12%	0	0.0%	0–8.8%*	<0.001^1^
Thickening of the nasopharynx	5	9.3%	3.08–20.3%	0	0.0%	0.06–13.16%	0.070^2^
Thickening of the nasal pyramid	11	20.4%	10.63–33.53%	0	0.0%	0–8.8%*	0.002^2^
Thickening of the soft palate	7	13.0%	5.37–24.9%	0	0.0%	0–8.8%*	0.019^2^
Erosion of the nasal bone	3	5.6%	1.16–15.39%	0	0.0%	0.8.8%*	0.259^2^

1 -Pearson Chi-square.

2 - Fisher's exact test,

*one-sided, 97.5% confidence interval.

**Table 4:** Opacification of the paranasal sinuses in the control and leishmaniasis groups.

Characteristics	Leishmaniasis		Control		P
	(n = 54)		(n = 40)		
**Partial opacification**					
1A (1%–33%)	46	85.2%	37	92.5%	0.344^2^
1B (34%–66%)	13	24.1%	0	0.0%	0.001^1^
1C (67%–99%)	10	18.5%	0	0.0%	0.004^2^
**Pansinus mucosal thickening**	23	42.6%	2	5.0%	<0.001^1^
**Total opacification**					
Complete opacification of at least one paranasal sinus	8	14.8%	0	0.0%	0.019^2^
Complete pansinus opacification	3	5.6%	0	0.0%	0.259^2^
Obliteration of the ostiomeatal complexes	13	24.1%	3	7.5%	0.035^1^

1 -Pearson Chi-square.

2 - Fisher's exact test.

### Demographic, clinical and radiological findings in the leishmaniasis group according to Lund-Mackay score

According to the cut-off value for chronic rhinosinusitis (score ≥4), 40/54 (74.1%) patients in the leishmaniasis group met the CT scan criteria for the disease [24], [25]. In the group with Lund-Mackay scores <4, most patients were female (64.3%) and had a mean age of 53±15 years. In contrast, in the group with Lund-Mackay scores ≥4, most patients were male (72.5%, p = 0.014) with a mean age of 62±12 years. Patients who presented higher levels of sinusopathy had mucosal lesions in more than one site (i.e., septum and palate), exhibited duration of disease for over two years before treatment and showed more severe disease at presentation. Rhinorrhea, epistaxis and alterations in nasal conchae were also associated with Lund-MacKay scores ≥4 (Table 5).

**Table 5:** Demographic, clinical and radiological findings of the leishmaniasis group according to the Lund-Mackay score.

Characteristics	Score<4		Score≥4		P
	(n = 14)		(n = 40)		
*Gender*					
Male	5	35.7%	29	72,5%	0.014^1^
**Duration of disease before 1^st^ treatment**					
<1 year	4	28.6%	5	12.5%	0.010^3^
1–2 years	7	50.0%	8	20.0%	
>2 years	3	21.4%	27	67.5%	
**Clinical severity of disease**					
Mild	13	92.9%	22	55.0%	0.014^3^
Moderate	1	7.1%	12	30.0%	
Severe	0	0.0%	6	15.0%	
**Anatomic site of disease**					
Septum	12	85.7%	39	97.5%	0.161^2^
Palate	3	21.4%	19	47.5%	0.088^1^
Larynx	2	14.3%	6	15.0%	1.000^2^
Septum and palate	2	14.3%	18	45.0%	0.041^1^
**Radiological findings (may be seen on physical exam)**					
Erosion of the nasal septum	7	50.0%	29	72.5%	0.188^2^
Collapse of the nasal pyramid	1	7.1%	7	17.5%	0.664^2^
Alterations of the nasal conchae	2	14.3%	19	47.5%	0.028^1^
**Symptoms**					
Nasal Obstruction	13	92.9%	40	100.0%	0.259^2^
Rhinorrhea	4	28.6%	24	60.0%	0.043^1^
Epistaxis	7	50.0%	34	85.0%	0.025^2^
Hyposmia	0	0.0%	8	20.0%	0.095^2^
Dyspnea	0	0.0%	3	7.5%	0.560^2^

1 -Pearson Chi-square,

2 -Fisher's exact test,

3 -Likelihood ratio.

In stepwise multiple regression analysis, the Lund-Mackay score was associated with the severity of disease at presentation (**β** = 3.38; p<0.001), epistaxis (**β** = 4.1; p<0.001) and rhinorrhea (**β** = 2.27; p = 0.018). The overall coefficient of determination (R^2^) of the model was 0.491.

## Discussion

Individuals with ML are usually described as adult patients who present mucosal metastases later in life due to a previous cutaneous lesion in childhood [5].

Clinical complications related to ML have already been described [10], but there is a lack of research on the prevalence of chronic rhinosinusitis in patients with the disease, whether in its active or inactive form, or in comparison with the general population. A recent multicentric study found a chronic rhinosinusitis prevalence of 10.9% in European countries [26]. In 2011, the prevalence of chronic rhinosinusitis in São Paulo was 5.51%, which represents more than 500,000 affected individuals [27].

**Figure 2:**
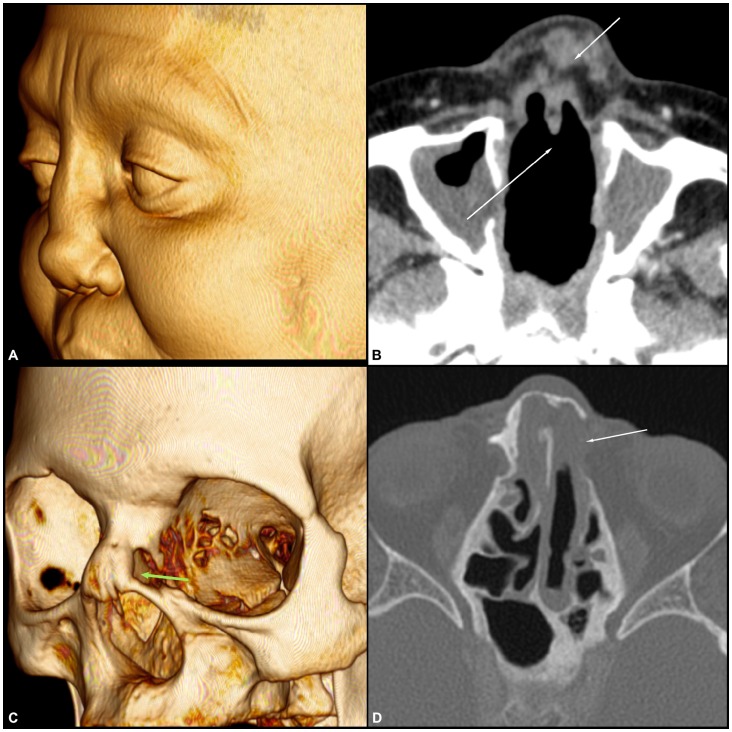
Volume-rendered 3D images of multi-slice CT data showing diffuse thickening of the nasal wings with collapse of the nasal pyramid (A) and erosion of the nasal bone (C). Axial computed tomography (CT) scan with soft tissue window (B) showing diffuse thickening of the nasal wings with collapse of the nasal pyramid, erosion of the nasal septum and opacification of the maxillary sinuses. Axial computed tomography (CT) scan of the bone window (D) demonstrates the erosion of the nasal bone and partial opacification of the ethmoid air cells.

**Figure 3:**
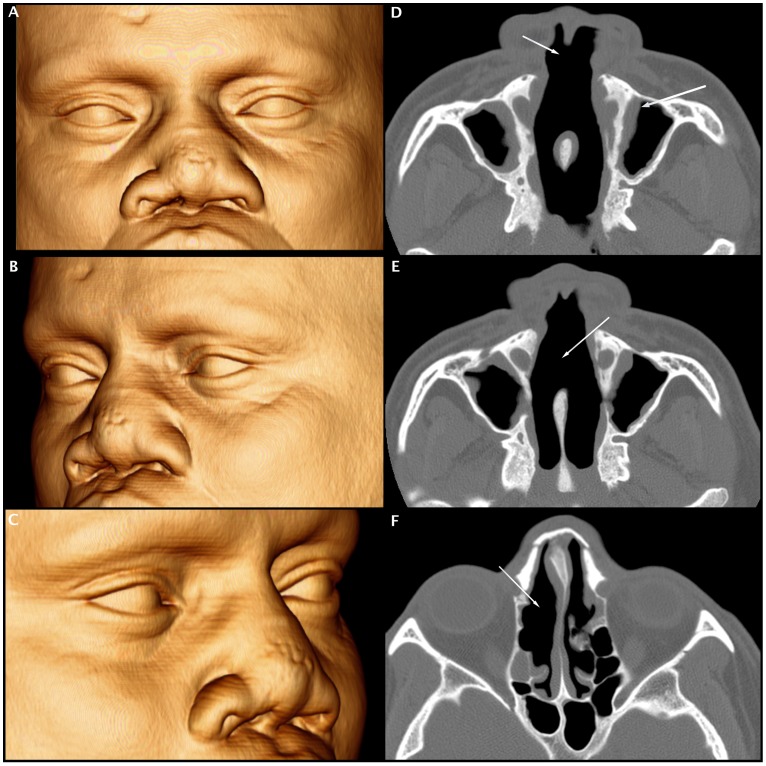
Volume-rendered 3D images of multi-slice CT data (A, B and C) and axial CT scans of the bone window (D, E and F) showing diffuse thickening of the nasal wings with collapse of the nasal pyramid (A, B and C). Axial CT scan shows erosion of the nasal septum (D and E), erosion of the anterior ethmoid cells (F) associated with mucous thickening of the nasal fossae (F), and partial opacification of the maxillary sinuses with thickening of their bony walls (osteitis-D and E).

**Figure 4:**
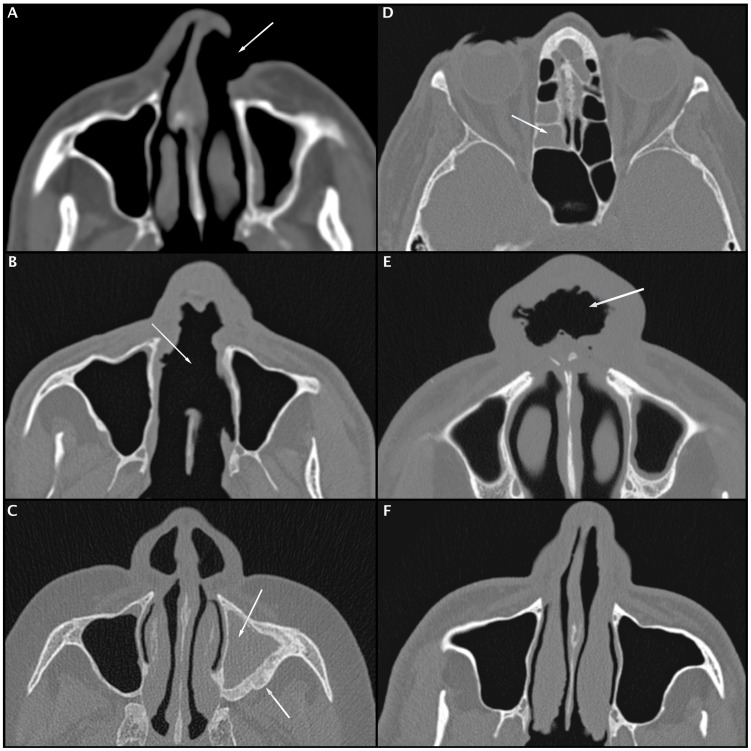
Axial CT scans of the bone window showing erosion of the left nasal wing (A), large erosion of the nasal septum (B), complete obliteration of the left maxillary sinuses with thickening of their bony walls (osteitis-C), complete obliteration of the ethmoid cells (D) and diffuse thickening of the nasal wings with collapse of the nasal pyramid (E). Axial CT scan shows the integrity of the nasal septum in a patient from the control group (F).

**Figure 5:**
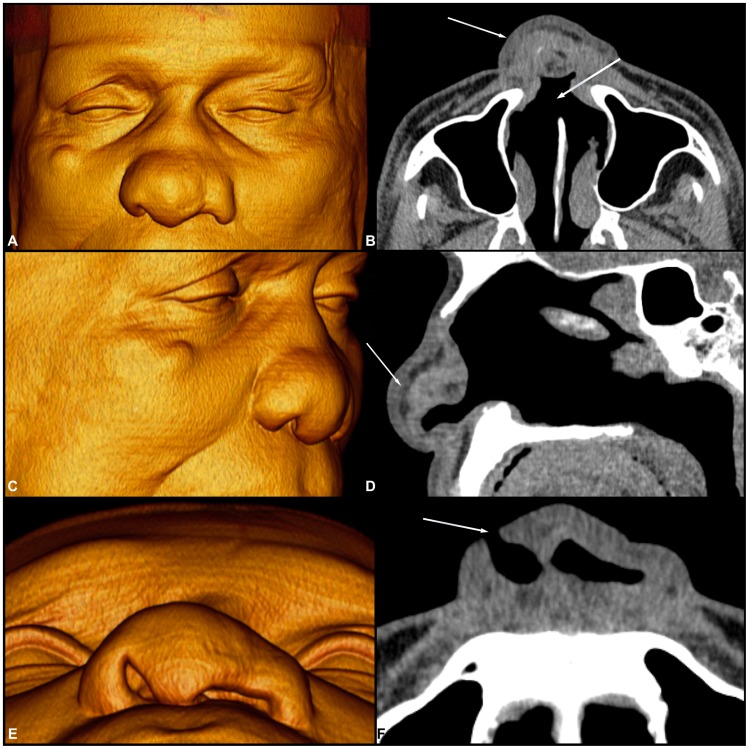
Volume-rendered 3D images of multi-slice CT data (A, C and E), axial CT scans (B and F) and a sagittal CT scan (D) with a soft tissue window showing diffuse thickening of the nasal wings and collapse of the nasal pyramid. Axial CT scan shows the erosion of the nasal septum (B and D) associated with diffuse thickening of the nasal wings and collapse of the nasal pyramid (B, D and F).

Several studies have reinforced the role of CT scans in the diagnosis and management of chronic rhinosinusitis [13], [28]. A CT scan of the paranasal sinus exhibits an excellent sensitivity (85%) and relatively good specificity (59%) with moderate to good accuracy (AUC value of 0.802) for the diagnosis of chronic rhinosinusitis when the Lund-Mackay score cut-off value is greater than or equal to four [24]. In the present study, patients with ML presented higher levels of chronic rhinosinusitis and significantly more osteitis of the paranasal sinus compared with the control group, which demonstrates the higher prevalence of sinus disease in this population, even after treatment [24], [25], [29]. These findings are similar to the results by Grindler et al. (2009) on the tomographic aspects of Wegener's granulomatosis [30]. CT scans of patients with ML also presented several structural alterations, such as erosion of the nasal septum; collapse of the nasal pyramid; thickening of the nasal cavity, nasopharynx, nasal pyramid and soft palate; and even erosion of the nasal bone, revealing a prominent destructive feature of the disease (Figures 2, 3, 4 and 5).

Within the ML group, multiple regression analysis showed that the Lund-Mackay score was associated with the severity of the disease at presentation, epistaxis and rhinorrhea. To increase clinical relevance, further analysis was performed, categorizing the Lunk-Mackay score with a cut-off of four, which tomographically defines sinusopathy (Table 5). Higher degrees of sinus disease were found among male individuals who had had the disease for more than two years before their first treatment and/or had a more severe presentation of ML. These variables may be considered predictive of chronic sinusitis in these patients. Interestingly, 14 patients in the ML group had a Lund-Mackay score <4, similar to the scores found in the control group. This may be explained by the fact that 13/14 (92.9%) of these patients had a milder presentation of the disease at diagnosis and 11/14 (78.6%) were diagnosed early (<2 years) (Table 5), reinforcing the importance of early diagnosis and treatment of this neglected disease.

CT scans have been used to evaluate the appearance, precise site, extension and clinical implications for structures surrounding head and neck granulomatous lesions, providing relevant data for an accurate diagnosis and treatment plan [31], [32]. Little is known about the exact location, pattern and extension of ML based on CT scans despite the damage caused to respiratory tract structures by this specific presentation of granulomatous disease.

**Figure 6:**
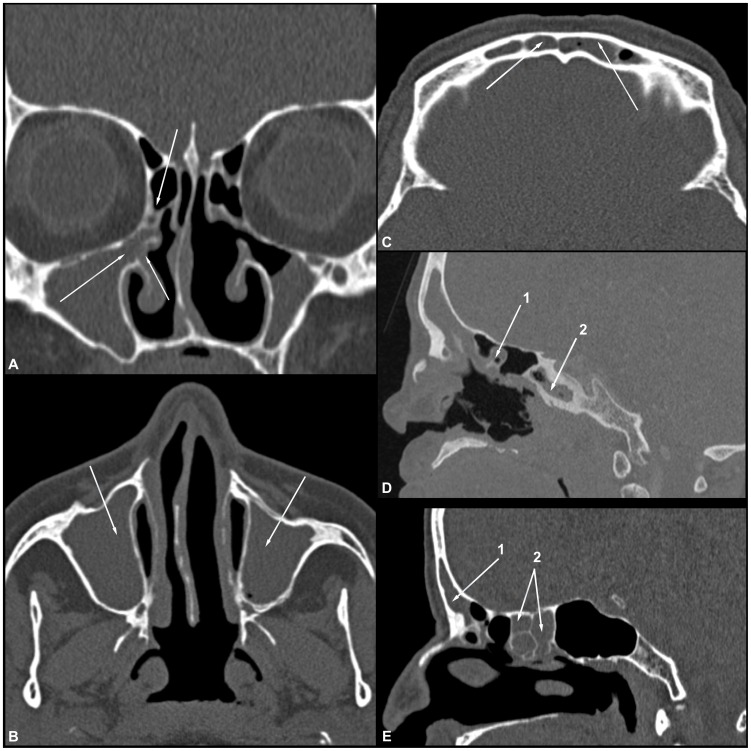
(A–E) Coronal, axial and sagittal CT images of the bone window demonstrate the opacification of the paranasal sinuses in a patient with treated mucosal leishmaniasis. Obliteration of the right ostiomeatal complex (A). Complete opacification of the maxillary sinuses (B) and frontal sinuses (C and E – arrow 1). Complete opacification of the sphenoid sinus (D – arrow 2) and ethmoid air cells (D – arrow 1 and E – arrow 2).

The findings in this study suggest that inflammation in patients with ML is not limited to the nasal mucosa but may extend to the paranasal sinuses and other upper respiratory tract structures. Any of the paranasal sinuses can be affected and patients frequently presented with involvement of multiple sinuses, although the pathophysiology of paranasal involvement in ML remains unclear (Figure 6). Mucosal thickening may be caused by a direct infection of the sinus mucosa by the *Leishmania* protozoan, which can live in the mucosa despite scar healing [33]. It may also be caused by chronic inflammation with continuous expression of tumor necrosis-**α** *in situ* after cicatrization of the mucosal lesion [34], or it may be secondary to the obstruction of the natural drainage pathways of the paranasal sinuses, leading to the stasis of secretions inside the sinuses and resulting in chronic sinusitis [7]. A fourth explanation may be related to the fact that the perforation of the nasal septum causes a swirling of the air at the time of its passage, changing its flow by providing greater kinetic energy relative to laminar flow and thereby increasing the exchange of heat and moisture in the nasal mucosa. This leads to a drop in the temperature and dryness of this region [35]–[37]. According to this hypothesis, the nasal mucosa is continuously damaged, leading to septal perforation-related symptoms such as nasal crusting, epistaxis, purulent drainage, nasal obstruction and nasal breathing with whistle [38]. Moreover, these disturbances in the physiology of the nasal mucosa may cause changes in the mucociliary epithelium, leading to the obstruction of drainage ostia and stasis of secretions within the sinuses and chronic sinusitis, as shown by Bhattacharyya (2007) [38]. These hypotheses seem to be simultaneously present, and all contribute to paranasal thickening.

Although the diagnosis and follow-up of patients with ML is often performed clinically, without the need for diagnostic imaging, the MDCT of patients with ML in our analysis showed several structural changes in the anatomy of the face, as previously described, indicating that the initial nasal impairment represents only the tip of the iceberg in this population. Though a complete otorhinolaryngologic examination (i.e., anterior rhinoscopy, oropharynx exam and a fiberoptic exam) is indispensable in patients with ML, it does not provide a more detailed assessment of the involvement of deeper osteocartilaginous structures [39]. Hence, in this scenario, the CT scan can be an interesting tool due to its proper assessment of structural alterations of bone and soft tissue structures of the face in the patients with ML. It also enables the visualization of the sinuses and their drainage pathways. In addition, MDCT provides objective evidence for the diagnosis and staging of chronic rhinosinusitis and an important “roadmap” to paranasal sinus anatomy whenever surgery is considered [24], [25].

Our study has potential limitations. We did not correct for multiple comparisons, leading to an increased risk of type I error and did not perform a formal sample size calculation, leading to an increased risk of type II error in some analyses and questionable precision in others. However, as the study focuses on a new field of investigation in this population, we assume it as an exploratory one and believe it still provides useful data and will generate hypotheses for future studies.

### Conclusion

The higher prevalence of chronic rhinosinusitis observed in CT scans of patients with treated ML in this study compared with the control group suggests that ML can be considered a risk factor for chronic rhinosinusitis in this population. The implications of these findings may be observed in everyday practice, where ML patients tend to require long-term follow-ups, representing direct health-care costs and several indirect losses in terms of impaired daily functioning [11].

In addition, MDCT can be considered a powerful assessment and follow-up tool for patients with ML due to the prompt identification of affected areas and its complementary role in nasofibrolaryngoscopy and, subsequently, early access to proper treatment. These outcomes lead to the improved management of an important disease that has traditionally been neglected.

## Supporting Information

Checklist S1 STROBE Checklist.
(DOCX)
