# Supplementary material for: Facial Structure Alterations and Abnormalities of the Paranasal Sinuses on Multidetector Computed Tomography Scans of Patients with Treated Mucosal Leishmaniasis
Source: PLoS Negl Trop Dis. 2014 Jul 31;8(7):e3001. doi: 10.1371/journal.pntd.0003001 (PMC4117457; doi:10.1371/journal.pntd.0003001)
Supplement: Checklist S1 — STROBE Checklist. (DOCX) [file pntd.0003001.s001.docx]

**STROBE CHECKLIST:** For manuscript # PNTD-D-14-00104R1

**Title:** Facial structure alterations and abnormalities of the paranasal sinuses on multidetector computed tomography scans of patients with treated mucosal leishmaniasis

| **Item** | **Item nº** | **Recommendation** | **Description of Manuscript Content** |
| --- | --- | --- | --- |
| **Title and Abstract** | 1 | Study design indicated with commonly used terms in title or abstract; Provide in abstract informative/balanced summary of methods and results | Study design is described in commonly used language in abstract (cross-sectional study with controls).  Basic results are provided regarding the use of multidetector computed tomography scans (MDCT) to assess the opacification of the paranasal sinuses in patients with treated mucosal leishmaniasis (ML) as well as any anatomical changes in the face associated with the disease. The results were compared with a control group. |
| **Introduction** |  |  |  |
| Background/ rationale | 2 | Explain the scientific background and the rationale for the study | The introduction summarizes the natural history of leishmaniasis and their clinical forms as well as its epidemiology. It also emphasizes the morbidity caused by the mucosal form as well as the lack of descriptive studies on the structural anatomical changes of the nose and paranasal sinuses in these patients using radiological methods. Pages 4-5. |
| Objectives | 3 | State objectives and pre-specified hypotheses | To determine the prevalence of chronic sinusitis using computed tomography (CT) of the sinuses in patients with ML post-treatment and to identify the existence of any tomographic changes in the nose and paranasal sinuses that may be related to ML. |
| **Methods** |  |  |  |
| Study design | 4 | Present key elements of study | Detailed steps of the study design are presented in chronological order. See pages 6–1^st^ paragraph of page 7, and pages 8-9. |
| Setting | 5 | Describe setting, relevant dates, recruitment, follow up and data collection | On pages 6 and 7, data regarding study setting and patient recruitment is provided. Data collection procedures, ethics statement, clinical findings, radiologic aspects and comparative analysis regarding ML and control groups are presented on pages 6-9. |
| **Item** | **Item nº** | **Recommendation** | **Description of Manuscript Content** |
| Participants | 6 | Cross sectional study with controls: provide eligibility criteria and sources and methods for the selection of participants | ML group: this prospective study evaluated the facial anatomy of 54 patients who had a confirmed diagnosis of ML and met the criteria for cure after treatment using MDCT of the paranasal sinuses. No patient had a history of rhinosinusitis, allergic rhinitis or sinus surgery, and no patients presented symptoms of infection of the upper airways on the day of the CT scan;  Control group: The control group was composed of 40 patients without a clinical diagnosis of ML who underwent MDCT of the orbit in 2009 and 2010, with sex and age distribution similar to the ML group. None of the control patients had a history of trauma or surgery of the skull, nose or paranasal sinuses prior to the day of CT scan, which was therefore assumed to show all paranasal sinuses. |
| Variables | 7 | Clearly define all outcomes, predictors, etc. | The main outcome is the percentage of chronic sinusitis in the patients of ML group (74.1%), as described on page 11. Patients from the ML group with a Lund-Mackay score ≥ 4 presented longer duration of disease before treatment and more severe presentation of the disease at diagnosis. These variables may be considered predictive of chronic sinusitis in those patients. |
| Data sources/ measurement | 8 | Provide sources of data and measurement methods | Analysis of the CT scans was performed from multiplanar reconstructions in the axial, coronal and sagittal views. The radiologist evaluated the degree of opacification of the paranasal sinuses (sinusopathy) and ostiomeatal complexes according to the Lund-Mackay criteria as well as the presence of any abnormality that could be related to leishmaniasis. Considering that incidental abnormalities are commonly observed in asymptomatic individuals, a Lund-Mackay score greater than or equal to four has been defined as the gold standard cut-off for chronic rhinosinusitis. |
| Bias | 9 | Describe efforts to assess sources of bias | The relatively small sample size and risk of bias due to convenience sampling may have limited the precision of the estimate of prevalence of chronic rinosinusitis in our population. However, our tertiary care Institution receives patients from different regions of Brazil and we examined |
| **Item** | **Item nº** | **Recommendation** | **Description of Manuscript Content** |
|  |  |  | contiguous patients followed in our outpatient facility, not only those with nasal or paranasal complaints. Given the high prevalence of radiographically diagnosed sinusitis in our study, we believe that ML patients are more prone to this complication than the general population. |
| Study size | 10 | Explain how the study size was arrived at | The Methods section describes the recruitment plan for patients in the ML and control groups. See pages 6 and first paragraph of page 7. As this was an exploratory study with convenience sample, no formal sample size calculation was performed. |
| Quantitative variables | 11 | Explain how quantitative variables were handled, grouped and the rationale for grouping | All tomographic findings in the ML and control groups were compared, along with the estimated grade of sinusopathy, according to the Lund-Mackay system. Data related to the ML group was further analyzed by dividing the 54 patients into 2 groups, according to the presence (Lund-Mackay ≥ 4) or absence (Lund-Mackay score < 4) of sinus disease, with 40 and 14 patients respectively. Considering that incidental abnormalities are commonly observed in asymptomatic individuals, a Lund-Mackay score greater than or equal to four has been defined as the gold standard cut-off for chronic rhinosinusitis. Exploratory univariate analyses were performed in order to identify predictive variables associated with a greater severity of sinusopathy in this population. |
| Statistical methods | 12 | Describe all statistical methods | Categorical variables were descriptively presented in tables containing absolute (n) and relative (%) values. The association between them was assessed using the chi-square test or likelihood ratio or Fisher's exact test. The normality of the quantitative variables was evaluated with the Kolmogorov-Smirnov test. Quantitative variables with normal distribution were presented descriptively in tables containing the mean and standard deviation. The averages of these variables were compared using the t-Student test. Quantitative variables without normal distribution were presented descriptively in tables containing the median and interquartile ranges. The distributions of these variables were compared using the Mann-Whitney test. P values ​​<0.05 were considered statistically significant. |

| **Item** | **Item nº** | **Recommendation** | **Description of Manuscript Content** |
| --- | --- | --- | --- |
| **Results** |  |  |  |
| Participants | 13 | Report n of participants at each stage of the study; Give reasons for non participation, Consider use of a flow diagram | ML group: this prospective study evaluated the facial anatomy of 54 patients who had a confirmed diagnosis of ML and met the criteria for cure after treatment using MDCT of the paranasal sinuses. No patient had a history of rhinosinusitis, allergic rhinitis or sinus surgery, and no patients presented symptoms of infection of the upper airways on the day of the CT scan;  Control group: A series of patients without a clinical diagnosis of ML who underwent MDCT of the orbit in 2009 and 2010 was extracted from a radiological examination database. None of the control patients had a history of trauma or surgery of the skull, nose or paranasal sinuses prior to the day of CT scan, which was therefore assumed to show all paranasal sinuses. A total of 40 patients with sex and age distributions similar to the mucosal leishmaniasis group were selected from the 269 patients who met the eligibility criteria. |
| Descriptive data | 14 | Give characteristics of study participants, indicate participants with missing data | ML group: 54 patients who had a confirmed diagnosis of ML and met the criteria for cure after treatment. The mean age of patients with ML in this study was 60 ± 13 (range 25–85) years. Sixty-three percent of patients were male, and 50% were Caucasian. The most common symptoms were nasal obstruction, epistaxis and rhinorrhea; high blood pressure was the most common comorbidity.  Control group: in the control group, the mean age was 55 ± 15 (range 28-90) years, and 62.5% of patients were male. Most patients in the control group were Caucasian (67.5%).  There were no participants with missing data in this study. |
| Outcome data | 15 | Cross sectional study: Report numbers of outcomes events or summary measures | CT scans in the ML and control groups demonstrated significant differences in terms of facial structure alterations (Table 3). Patients from the ML group showed more severe levels of partial opacification and pansinus mucosal thickening (23/54, 42.6%) and a greater severity of total opacification (Table 4). In general, patients from this group presented a higher median Lund-Mackay score (7, p<0.001) than did patients from the control group (3, p<0.001). |
| **Item** | **Item nº** | **Recommendation** | **Description of Manuscript Content** |
| Main results | 16 | Report n of participants at each stage of the study; Give reasons for non participation, Consider use of a flow diagram | ML group: this prospective study evaluated the facial anatomy of 54 patients who had a confirmed diagnosis of ML and met the criteria for cure after treatment using MDCT of the paranasal sinuses. No patient had a history of rhinosinusitis, allergic rhinitis or sinus surgery, and no patients presented symptoms of infection of the upper airways on the day of the CT scan;  Control group: A series of patients without a clinical diagnosis of ML who underwent MDCT of the orbit in 2009 and 2010 was extracted from a radiological examination database. None of the control patients had a history of trauma or surgery of the skull, nose or paranasal sinuses prior to the day of CT scan, which was therefore assumed to show all paranasal sinuses. A total of 40 patients with sex and age distributions similar to the mucosal leishmaniasis group were selected from the 269 patients who met the eligibility criteria. |
| Other analyses | 17 | Report on other analyses conducted (sub group, etc) | According to the cut-off value for chronic rhinosinusitis (score ≥4), 40/54 (74.1%) patients in the leishmaniasis group met the CT scan criteria for the disease. In the group with Lund-Mackay scores <4, most patients were female (64.3%) and had a mean age of 53 ±15 years. In contrast, in the group with Lund-Mackay scores ≥4, most patients were male (72.5%, p=0.014) with a mean age of 62 ±12 years. Patients who presented higher levels of sinusopathy had mucosal lesions in more than one site (i.e., septum and palate), exhibited duration of disease for over two years before treatment and showed more severe disease at presentation. Rhinorrhea, epistaxis and alterations in nasal conchae were also associated with Lund-MacKay scores ≥4. |
| **Discussion** |  |  |  |
| Key results | 18 | Summarize key results with reference to study objectives | Key results are summarized in the three paragraphs of the Discussion section (page 12). |

| **Item** | **Item nº** | **Recommendation** | **Description of Manuscript Content** |
| --- | --- | --- | --- |
| Limitations | 19 | Discuss limitations of the study, including possible biases, imprecision and direction and magnitude of such | We assume our study as an exploratory one. We did not correct for multiple comparisons, leading to an increased risk of type I error, and did not define a primary endpoint or performed a formal sample size calculation, leading to an increased risk of type II error. However, as the study focus on a new field of investigation in this population, we believe it is still provides useful data for future studies. |
| Interpretation | 20 | Give a cautious overall interpretation considering objectives, results form similar studies and other relevant evidence | The higher prevalence of chronic rhinosinusitis observed in CT scans of patients with treated ML in this study compared with the control group suggests that ML can be considered a risk factor for chronic rhinosinusitis in this population. The CT scan can be an interesting tool due to its proper assessment of structural alterations of bone and soft tissue structures of the face in the patients with ML. It also enables the visualization of the sinuses and their drainage pathways. In addition, MDCT provides objective evidence for the diagnosis and staging of chronic rhinosinusitis and an important “roadmap” to paranasal sinus anatomy whenever surgery is considered. |
| Generalizability | 21 | Discuss the generalizability (external validity) of the study results | External validity may be questioned by the study design, mainly due to the use of a convenience sample. But the high prevalence of sinus disease that was detected with an accurate method of diagnosis makes our results evident and we believe chronic sinusitis should be a concern in patients with ML from other populations. |
| **Other information** |  |  |  |
| Funding | 22 | Provide source of funding and role of funders | No funding supported this project. |
